# Holistic View of ALK TKI Resistance in ALK-Positive Anaplastic Large Cell Lymphoma

**DOI:** 10.3389/fonc.2022.815654

**Published:** 2022-02-08

**Authors:** Yuan Wang, Jing He, Manyu Xu, Qingfeng Xue, Cindy Zhu, Juan Liu, Yaping Zhang, Wenyu Shi

**Affiliations:** ^1^Department of Oncology, Affiliated Hospital of Nantong University, Nantong, China; ^2^Nantong University School of Medicine, Nantong, China; ^3^Department of Clinical Biobank, Affiliated Hospital of Nantong University, Nantong, China; ^4^Department of Psychology, University of California, Los Angeles (UCLA), Los Angeles, CA, United States; ^5^Department of Hematology, Affiliated Hospital of Nantong University, Nantong, China

**Keywords:** ALCL, ALK, ALK-TKI, lymphoma, drug resistance, therapy

## Abstract

Anaplastic lymphoma kinase (ALK) is a receptor tyrosine kinase expressed at early stages of normal development and in various cancers including ALK-positive anaplastic large cell lymphoma (ALK+ ALCL), in which it is the main therapeutic target. ALK tyrosine kinase inhibitors (ALK TKIs) have greatly improved the prognosis of ALK+ALCL patients, but the emergence of drug resistance is inevitable and limits the applicability of these drugs. Although various mechanisms of resistance have been elucidated, the problem persists and there have been relatively few relevant clinical studies. This review describes research progress on ALK+ ALCL including the application and development of new therapies, especially in relation to drug resistance. We also propose potential treatment strategies based on current knowledge to inform the design of future clinical trials.

## 1 Introduction

Anaplastic large cell lymphoma (ALCL) is an aggressive cluster of differentiation (CD)30+ peripheral T-cell lymphoma (PTCL) that accounts for approximately 10%–15% of pediatric and 1%–2% of adult non-Hodgkin lymphoma (NHL) cases (1). Over 90% of children and adolescents with ALCL are ALK positive (ALK+) while the rate among adult patients is 40%–50% (2). The main feature of ALK+ ALCL is the expression of ALK fusion proteins such as nucleophosmin (NPM)–ALK, TNF receptor-associated factor (TRAF)1–ALK, 5-aminoimidazole-4-carboxamide ribonucleotide formyltransferase/IMP cyclohydrolase ATIC–ALK, ring finger protein (RNF)213–ALK, and tropomyosin (TPM)3–ALK (3–6). NPM–ALK is the most prevalent of these fusions and is detected in 75%–80% of adults and 90% of children with ALK+ ALCL (7, 8). NPM–ALK arises from the fusion of the *ALK* gene on chromosome 2p23 with the *NPM* gene on chromosome 5q35. ALK is normally expressed in cells of the small intestine, testis, and colon but not in lymphocytes (9). In ALK+ ALCL, NPM–ALK is highly expressed as a result of a high copy number of the *NPM* promoter and constitutively activates NPM–ALK and downstream signaling including signal transducer and activator of transcription (STAT)3, phospholipase (PLC)γ, phosphatidylinositol 3-kinase (PI3K)–protein kinase B (AKT), and mitogen-activated protein kinase (MAPK)–extracellular signal-regulated kinase (ERK) pathways that are important for cell survival and proliferation, metabolic transformation, and immune evasion *via* the oligomerization of NPM (10). Therefore, NPM–ALK is a target of therapeutic strategies in ALK+ ALCL. Alectinib was approved in Japan for the treatment of children and adults with relapsed/refractory (R/R) NPM–ALK+ ALCL, while crizotinib has been approved for this indication by the US Food and Drug Administration. However, a subset of patients responds poorly to ALK TKIs due to mutation/amplification of the *ALK* gene, which reduces sensitivity to these drugs. Tumors also survive by other mechanisms such as autophagy, anti-apoptosis, and ALK bypass substitution, among others.

## 2 Signaling Pathways in ALK+ ALCL

### 2.1 STAT3 Pathway

**Figure 1:**
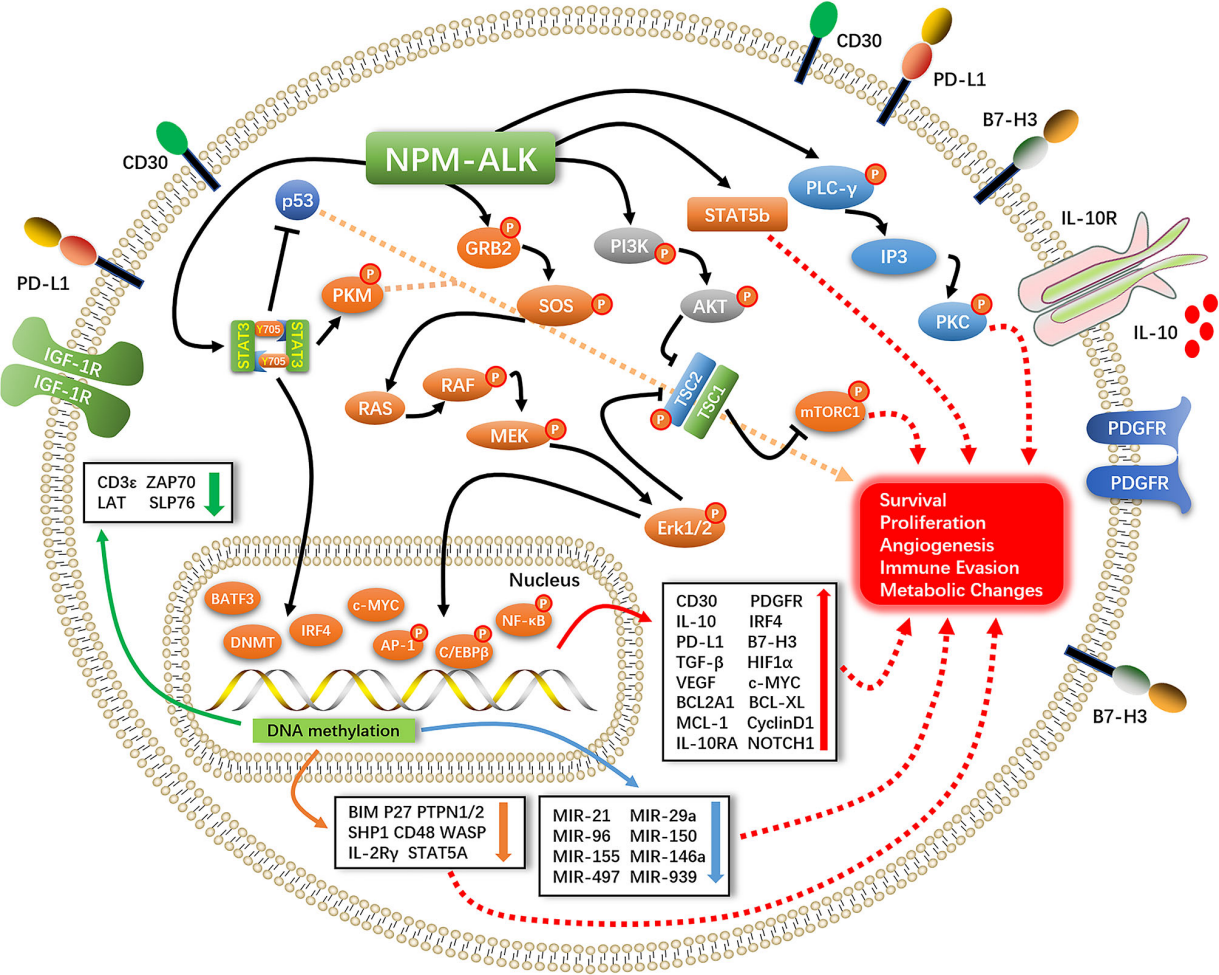
Pathogenesis of ALK+ALCL.

STAT3 is a downstream effector of ALK that plays an important role in promoting cell survival, proliferation, and immune evasion (11). STAT3 exerts anti-apoptotic effects in ALK+ ALCL mainly by upregulating the anti-apoptotic protein B cell lymphoma extra-large (Bcl-xL) and antagonizing the tumor suppressor P53 (12). STAT3-induced expression of transforming growth factor (TGF)-β, interleukin (IL)-10, and programmed death ligand (PD-L1,CD274, B7-H1) creates a tumor-suppressive microenvironment (13–15). STAT3 directly binds to the promoter of hypoxia-inducible factor (HIF)-1α to promote gene expression; HIF-1 α in turn induces the expression of vascular endothelial growth factor (VEGF) and promotes tumor angiogenesis (16, 17). STAT3 also plays an important role in epigenetic regulation of gene expression. Under normal conditions, NPM–ALK phosphorylates the Y405 residue of STAT3, which causes the dimerization of phosphorylated STAT3 and its translocation into the nucleus where it modulates gene transcription in a methylation-dependent manner (18, 19). This leads to the silencing of oncogenes such as STAT5A, IL-2 receptor gamma (IL-2Rγ), Bcl-2-like protein 11 (BIM), protein tyrosine phosphatase non-receptor type 6 (SHP1), and CD48 (20–25) and suppresses the expression of micro (mi)RNAs with oncogenic effects such as miR-150, miR-497, miR-21, miR-29a, miR-939, miR-96, miR-155, and miR-146a (26–31). The consequent silencing of T-cell receptor-related genes including CD3ϵ, zeta-chain-associated protein kinase (ZAP)70, linker for activation of T cells (LAT), and SH2 domain-containing leukocyte protein of 76 kDa (SLP76) results in the loss of T cell identity (32). It was recently reported that NPM–ALK mediates STAT3 acetylation to inhibit the expression of tumor suppressor genes; inhibiting STAT3 acetylation resulted in their re-expression and ALCL cell apoptosis (33) (**Figure 1**).

### 2.2 PLC-γ Pathway

Activation of NPM–ALK leads to PLC-γ tyrosine phosphorylation (Y783, Y1254) and induction of its catalytic activity, leading to the breakdown of phosphatidylinositol 4,5-bisphosphate (PIP2) to inositol trisphosphate (IP3) and activation of protein kinase (PK)C; this process plays an important role in NPM–ALK-mediated mitogenic signaling (34–36) (**Figure 1**).

### 2.3 PI3K–AKT Pathway

NPM–ALK interacts with the SH2/SH3 domain of the PI3K regulatory subunit (p85) (37). P85 binds to the catalytic subunit (p110) and converts PIP2 to phosphatidylinositol (3,4,5)-trisphosphate (PIP3), which binds to the pleckstrin homology domain of AKT and facilitates its translocation from the cytoplasm to the cell membrane. T308 and S473 phosphorylation by phosphatidylinositol-dependent protein kinase (PDK)1 and PDK2 activates AKT (38–40), mammalian target of rapamycin (mTOR) is then phosphorylated and activated by AKT to promote the survival and proliferation of ALK+ ALCL cells (41) (**Figure 1**).

### 2.4 MAPK Kinase (MEK)–ERK Pathway

MEK–ERK1/2 signaling stimulates cell proliferation by promoting cell cycle progression *via* the protein-dependent kinase (CDK)4 and retinoblastoma protein (RB) pathways. CDK4 binds to cyclin D1 to phosphorylate Rb and release the transcription factor E2F, which induces the expression of CDK4 as well as cyclin D1, leading to the entry of cells into S phase; it also promotes cell survival and proliferation *via* ERK1/2–mTOR signaling (42, 43). ERK1/2 phosphorylates JUNB, a member of the activator protein (AP)-1 family and an important transcription factor in ALK+ ALCL. JUNB transcriptional targets have been shown to be involved in cell proliferation, anti-apoptosis, immune evasion, and immune phenotype transition (44, 45) (**Figure 1**).

## 3 Mechanisms of ALK TKI Resistance

**Figure 2:**
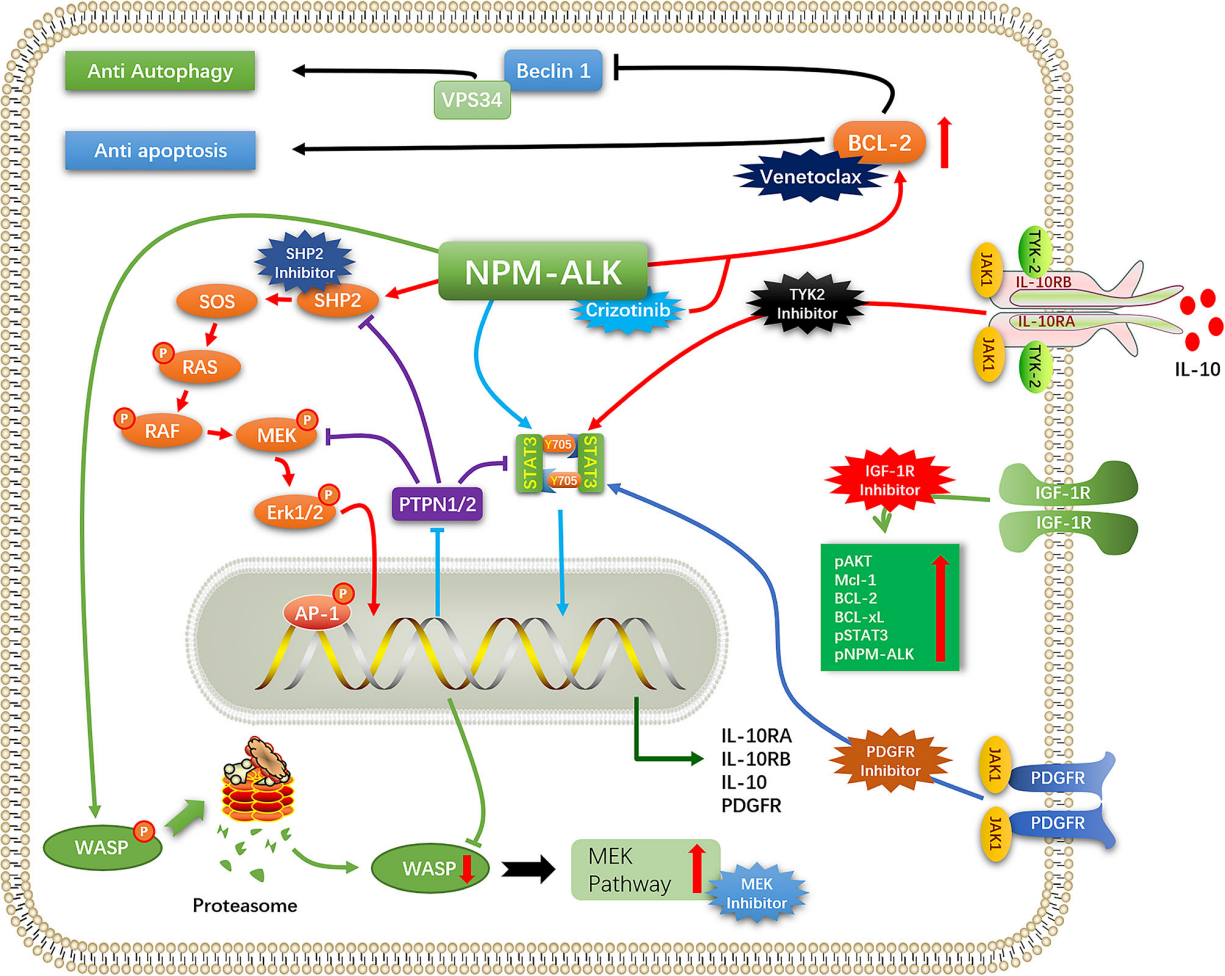
Mechanisms of resistance to ALK-TKI.

ALK+ ALCL treated with the chemotherapy regimen consisting of cyclophosphamide, doxorubicin hydrochloride (hydroxydaunorubicin), vincristine sulfate, and prednisone (CHOP) has a favorable clinical outcome, although the rate of recurrence is high. Patients who relapse after first-line chemotherapy usually have a poor prognosis, while some patients are inherently resistant to chemotherapy regimens such as CHOP. ALK TKIs can improve the prognosis of patients with R/R ALK+ ALCL. In a clinical trial of children with R/R ALK+ ALCL treated with crizotinib (NCT00939770), the objective response rate (ORR) was 90% and the complete response (CR) rate was 80% (46). In a phase 2 clinical trial of alectinib for the treatment of R/R ALK+ ALCL(Range, 6-70 years), the ORR after alectinib treatment was 80% and the CR rate was 60%, while the 1-year PFS, event-free survival (EFS), and overall survival (OS) rates were 58.3%, 70.0%, and 70.0% respectively (47). But a new problem has arisen: Among patients who have a high response to crizotinib monotherapy, about 30-40% of patients have developed further resistance to the drug (48). ALK mutation/amplification reduces sensitivity to ALK TKIs, necessitating a switch to another drug. Furthermore, tumors mitigate the cytotoxicity of ALK TKIs through a variety of mechanisms (ALK bypass substitution, autophagy, anti-apoptosis, etc), thereby reducing/eliminating their dependence on ALK signaling and promoting tumor cell survival. In this situation, even complete inhibition of ALK cannot prevent tumor progression, and combination therapy or targeting of proteins other than ALK (eg, CD30, B7-H3, heat shock protein [HSP]90, etc) may be necessary (**Figure 2**).

### 3.1 ALK-Related Resistance

#### 3.1.1 ALK Mutation

ALK mutations can lead to the development of ALK resistance through a variety of mechanisms such as by blocking the binding of ALK TKI to the ATP-binding site of ALK (L1196M, I1171T/N/S, G1269A, G1202R/del, G1202N, S1206Y/C, etc) (47, 49–54). Several point mutations in the structural domain of NPM–ALK kinase have been identified in ALK+ ALCL (L1122V, L1196M, F1174V, L1198F, P1139S, and G1202R) that have been implicated in the development of resistance to ALK TKIs (55). For example, the F1174V/L1198F and L1196M/D1203N double mutations were shown to confer higher resistance to the inhibitors (56).

#### 3.1.2 ALK Amplification

ALK+ ALCL tumor cells also develop ALK TKI resistance through amplification of NPM–ALK, which was shown to be overexpressed in ALK+ALCL-resistant cell lines (48, 55, 57, 58).

### 3.2 ALK-Independent Drug Resistance

#### 3.2.1 Activation of Bypass Signaling Pathways

IL-10 signaling bypass leads to crizotinib resistance (59). In ALK+ ALCL, IL-10 promotes the proliferation of ALK+ ALCL and reduces the sensitivity of ALCL cells to ALK TKI. A screen of tumors from ALK+ ALCL patients who progressed within 3 months of crizotinib treatment found that IL-10RA was overexpressed in tumor cells, leading to ALK TKI resistance. In resistant ALK+ ALCL cell lines, autocrine IL-10/IL-10RA signaling activated tyrosine kinase (TYK)2/Janus kinase (JAK)1 signaling instead of the ALK–STAT3 pathway to activate STAT3, which stimulated the expression of IL-10, IL-10RA, and IL-10RB. Thus, the strength of IL-10RA signaling in ALK+ ALCL affects tumor sensitivity to crizotinib. IL-10 is a known immunosuppressive factor that directly inhibits effector T cells, promotes T cell exhaustion, and inhibits T cell activation through myeloid-derived suppressor cells and induction of regulatory T cells (60, 61). This could allow ALK+ ALCL to achieve immune evasion and may result in the development of drug resistance, although there have been no studies investigating this possibility.

Activation of insulin-like growth factor 1 receptor (IGF-1R) signaling is another mechanism of ALK resistance. Type 1 IGF-1R is commonly expressed in ALK+ ALCL. In a crizotinib resistance model, IGF-1R signaling was shown to be increase with drug concentration and reduced the cytotoxicity of crizotinib; conversely, crizotinib combined with IGF-1R inhibitors partly restored the sensitivity of resistant cells (62). ALK phosphorylates IGF-1R at the C-terminal Y338 residue; in turn, IGF-1R increases the phosphorylation of ALK and its downstream effectors and promotes the survival of ALK+ ALCL cells by increasing the expression of anti-apoptotic proteins (myeloid cell leukemia [Mcl]-1, Bcl-2, Bcl-xl, etc) (63). The combined use of IGF-1R inhibitors and ALK TKIs can suppress ALK and downstream signaling, thereby enhancing the sensitivity of ALK+ ALCL to ALK TKIs and reducing crizotinib resistance caused by activation of the IGF-1R pathway, making combination therapy a potential treatment regimen. (64).

PDGFR expression and activation is a key driver of ALCL proliferation, survival and spread. NPM-ALK promoted the expression of PDGFRB through NPM-ALK/AP-1 (JUN/JUNB) (65). High expression of PDGFRB could also be seen in most ALK+ALCL. PDGFR promoted cell survival *via* JAK1/STAT3 and AKT(PKB)/mTOR pathways (66, 67). The level of PDGFR-mRNA decreased after application of PDGFR inhibitors, indicating that PDGFR is promoting its own expression, ALK-TKI in combination with PDGFR inhibitors reduces lymphoma growth and decreases relapse rates, and is a potential treatment option for patients with drug-resistant lymphoma. (65).

Activation of the MEK pathway also contributes to ALK TKI resistance, mainly through deficiency/low expression of Wiskott–Aldrich syndrome protein (WASP) (68), a tumor suppressor whose loss leads to tumor development and invasion (69). NPM–ALK negatively regulates the expression of WASP by directly phosphorylating Y102, leading to proteasome-mediated protein degradation and preventing protein binding to WASP-interacting protein (WIP) (70). NPM–ALK was also shown to repress WASP protein expression *via* STAT3–CCAAT/enhancer-binding protein (C/EBP)-β signaling. Downregulation of WASP was shown to enhance MEK pathway activation in ALK+ALCL. NPM–ALK can form a complex with the guanine exchange factor VAV1 to enhance the activity of cell division cycle (CDC)42, which promotes the progression of ALK+ ALCL; meanwhile, WASP inhibits the binding of CDC42 to GTP (71). Protein tyrosine phosphatase (PTPN)1/2 expression was found to be downregulated in ALK+ ALCL, leading to the overactivation of MEK, SHP2, and Janus kinase (JAK)/STAT and resistance to ALK TKIs (72).

Although ALK inhibition undermines the survival and proliferation of ALCL cells, not all cellular changes caused by ALK expression are reversed. For example, IL-10R, WASP, and MEK signaling was not downregulated by application of crizotinib; instead, tumor cells developed resistance to the drug through autocrine IL-10 secretion by ALK+ ALCL cells and activation of IL-10R signaling, which replaced the role of ALK in promoting IL-10R expression, suggesting that cytologic changes caused by ALK led to the emergence of ALK-independent mechanisms of tumor cell survival (59, 68). Some oncogenes that were downregulated *via* epigenetic modifications were not upregulated by crizotinib, but were instead involved in crizotinib resistance, suggesting that the decrease in their expression was not reversed (68, 72) (**Figure 2**).

#### 3.2.2 Autophagy and Apoptosis

Autophagy activation and inhibition play different roles according to the tumor type. In ALK+ ALCL, inhibition of autophagy facilitated tumor survival in the presence of ALK TKIs (73). Crizotinib was shown to decrease autophagic flux in ALK+ ALCL, which was important for tumor survival of NPM–ALK+ ALCL; this cytoprotective response reduced the cytotoxicity of crizotinib (56, 74). Rapamycin, an mTOR inhibitor that activates autophagy, reduces the survival of ALCL cells (75). The expression level of miR-7-5P was found to be downregulated in ALK+ ALCL by the application of crizotinib; it was later shown that miR-7-5P directly targets the 3′ untranslated region of RAF1 transcript, thereby negatively regulating RAF1 expression and reducing the inhibitory phosphorylation of Unc-51–like autophagy-activating kinase (ULK)1 (S757) to promote autophagy. Conversely, downregulation of miR-7-5P following crizotinib application inhibited autophagy. Potentiating the effect of miR-7-5P using an miRNA mimic enhanced crizotinib-induced autophagic flux and cytotoxicity (76). Interestingly, in ALK+ ALCL stem cells, crizotinib more potently induced autophagy and enhanced tumor cell resistance to the drug, an effect mediated by MYC (77).

Elevated levels of Bcl-2 have been observed upon treatment with crizotinib. Bcl2 is anti-apoptotic protein that inhibits autophagy, which can lead to drug resistance in tumors. Inhibition of Bcl-2 significantly increased crizotinib-induced autophagy (78, 79).

Decreased autophagic flux in ALK+ ALCL plays a role in drug resistance, and tumor cells are regulated in various ways that lead to inhibition of autophagy and crizotinib resistance including regulation of miRNAs and Bcl-2 expression. Autophagy inhibition can reverse the crizotinib-induced decrease in cell viability, thus limiting the cytotoxicity of crizotinib (**Figure 2**).

## 4 Strategies to Overcome ALK TKI Resistance

### 4.1 ALK Sequencing Analysis

For patients with ALK TKI resistance, tissue biopsy and pathologic examination can reveal the mechanism of resistance and inform clinical decisions, especially drug selection. For example, ALK TKIs can be selected that target specific ALK mutations. On the other hand, the absence of ALK mutation can indicate the development of resistance by the tumor, requiring ALK TKI combination therapy. For highly resistant (especially compound) mutations, ALK amplification may not be detected or the cause of resistance may not be clear, in which case switching ALK TKIs or using combination therapy may not be effective and therapeutic targets other than ALK should be considered. The development of ALK sequencing has aided the precision treatment of ALK+ ALCL and has revealed novel drug resistance mutations; the elucidation of ALK TKI resistance mechanisms will facilitate the development of new treatment strategies.

### 4.2 Intermittent Treatment

**Figure 3:**
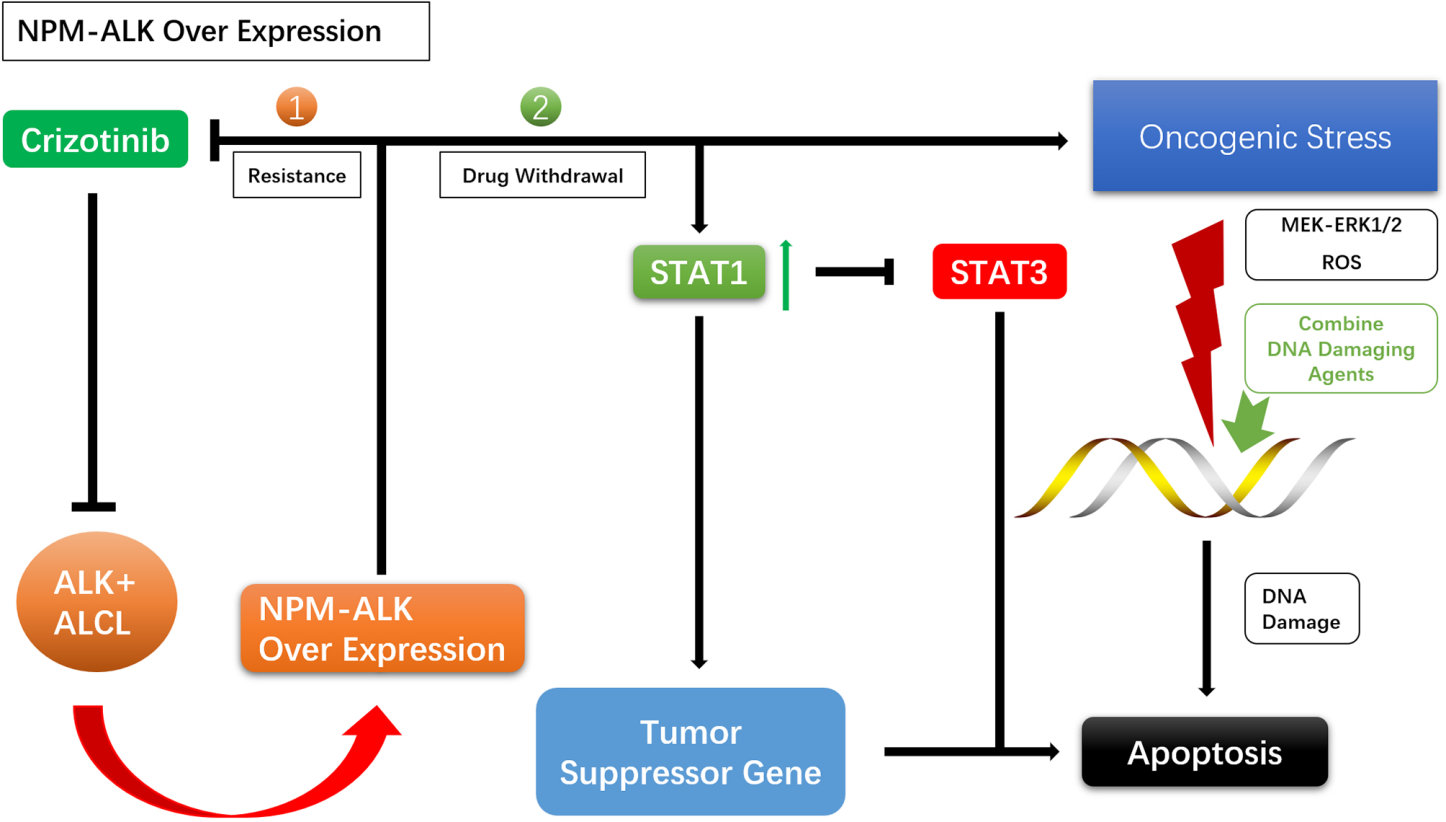
Mechanisms of intermittent treatment.

For drug resistance caused by NPM–ALK overexpression, crizotinib discontinuation can lead to overactivation of ALK oncogenic signaling and enhanced mitochondrial activity, reactive oxygen species (ROS) production, and activation of the MEK–ERK1/2 pathway, causing DNA damage. This may result in apoptosis of ALK TKI-resistant cells and the resensitization of tumor cells to crizotinib (56, 57, 80–83). NPM–ALK overexpression promotes STAT1 phosphorylation, and high levels of phosphorylated (p)STAT1 antagonize STAT3 and activate tumor suppressor genes, promoting cell death. Enhanced NPM–ALK expression following withdrawal of ALK TKI leads to the upregulation of pSTAT1, which antagonizes STAT3 and induces apoptosis (84, 85). Given the damaging effects of excessive tumorigenic signals on DNA, agents that Enhance DNA damage and inhibit DNA repair can be used during drug withdrawal to enhance the effects NPM–ALK (**Figure 3**).

### 4.3 Novel Drugs Targeting Resistant ALK+ ALCL

#### 4.3.1 Novel ALK TKIs

Second-generation ALK TKIs such as alactinib, ceritinib, brigatinib, lorlatinib, and ZX-29 have more potent activity in the central nervous system than crizotinib and can overcome the effects of most crizotinib resistance mutations (86) (**Table 1**). Alectinib is an orally administered drug with greater potency than crizotinib that was shown to be effective in crizotinib-resistant tumors (100). including ALK+ non-small cell lung cancer (NSCLC), with neutropenia and elevated levels of creatine kinase as the most serious adverse effects (101, 102). Ceritinib is a small molecule ALK TKI that has demonstrated efficacy in ALK-positive tumors including ALCL, inflammatory myofibroblastic tumor, neuroblastoma, and rhabdomyosarcoma; in ALK+ ALCL, the most commonly reported complication was elevated transaminases (103). Brigatinib is an ALK/ROS1 inhibitor with higher selectivity than alectinib or ceritinib that can overcome most crozotinib resistance mutations including G1202R, with an ORR of 74%, median PFS of 14.5 months, and 1-year OS rate of 83% in crizotinib-resistant patients (96, 104). Brigatinib has shown superior efficacy to crizotinib in the first-line setting, and an ongoing study is investigating brigatinib in ALCL (105). However, second-generation ALK TKIs are more likely to lead to the development of resistance than crizotinib (54). The third-generation ALK TKI loratinib overcomes nearly all single resistance mutations to second-generation inhibitors and is more potent than brigatinib against the G1202R mutation. However, G1202R was shown to reduce the sensitivity of tumor cells to brigatinib and loratinib, and tumor cells harboring double mutations (D1203N+E1210K and F1174C+D1203N) had lower sensitivity to loratinib, whereas none of the second-generation ALK TKIs were effective (54). ZX-29 is a novel ALK TKI that induces apoptosis by stimulating the production of ROS, overcoming the drug resistance conferred by the ALK G1202R mutation and exhibiting greater cytotoxicity than ceritinib (106, 107). The Fms-related receptor tyrosine kinase (FLT)3/AXL inhibitor gilteritinib is currently mainly used for R/R acute myeloid leukemia (108). Gilteritinib inhibits not only wild-type and mutant ALK but also overcomes highly resistant double mutations (I1171N+F1174I, I1171N+L1198H, and others containing I1171N) as well as single mutations by forming hydrogen bonds with the ALK E1197, M1199, and E1210 residues (99, 109). Mutations such as L1196M, I1171T/N/S, G1269A, G1202R/del, G1202N, and S1206Y/C that sterically hinder the binding of ALK TKIs to ALK are a common resistance mechanism (47, 49–54). Gilteritinib inserts into the ATP-binding site of ALK and therefore has an advantage over the first 3 generations of ALK TKI in terms of drug resistance, providing an additional treatment option for patients. Gilteritinib has also demonstrated efficacy against ALK+ ALCL in preclinical studies (110).

**Table 1:** Resistant and sensitive mutations of ALK-TKIs.

ALK-TKI	Crizotinib	Ceritinib	Alectinib	Brigatinib	Lorlatinib	Gilteritinib
**Sensitive** **mutations**	**L1198F** **C1196Y+L1198F** **C1156Y+L1198F**	**G1269A/S** **I1171T/N** **L1196M** **S1206C/Y** **I1171S+G1269A**	**C1156Y/T** **F1174C/L/V** **G1269A/S** **I1151Tins** **L1196M** **L1256F** **L1152P/R** **S1206C/Y** **I1171N+L1256F**	**C1156Y/T** **E1210K** **F1174C/L/V** **F1245A** **G1202R(-)** **G1269A/S** **I1151tins** **L1152P/R** **L1196M** **I1171S + G1269A**	**C1156Y/T** **E1210K** **F1174C/L/V** **G1269A/S** **G1202R** **I1151Tins** **L1152P/R** **L1196M** **S1206C/Y**	**I1171N** **I1171N+F1174I** **I1171N+L1198H**
**Resistance mutations**	**C1156Y/T** **E1210K** **F1245V** **G1269A/S** **G1202R** **I1151Tins** **I1171N/S/T** **L1196M** **L1152P/R** **S1206Y/C** **V1180L** **E1210K +D1203N** **L1196M+G1202R**	**C1156Y/T** **F1174C/L/V** **I1151Tins** **G1202R** **V1180L** **I1171N+F1174I** **I1171N+L1198H** **L1196M+G1202R**	**G1202R** **I1171T/N/S** **V1180L** **L1196M+G1202R**	**D1203N** **G1202R(-)** **I1171T/N/S** **V1180L** **L1196M+G1202R**	**C1156F/Y** **L1198F** **L1256F** **G1202R+L1196M** **C1196Y+L1198F** **G1202R+T1151M** **G1202R+F1174C** **I1171N+F1174I** **I1171N+L1198H** **L1196M/D1203N** **F1174L/G1202R** **C1156Y/G1269A**	
**References**	(54, 87–89)	(54, 88–91)	(54, 88, 89, 92, 93)	(87–89, 94–96)	(56, 87–89, 91, 97, 98)	(99)

ALK kinase structural domain mutations common to NSCLC and ALCL have been identified. **Table 1** summarizes mutations known to confer resistance/sensitivity to ALK TKIs that have potential application in the treatment of ALK+ ALCL (**Table 1**).

#### 4.3.2 Proteolysis-Targeting Chimera (PROTAC)

**Figure 4:**
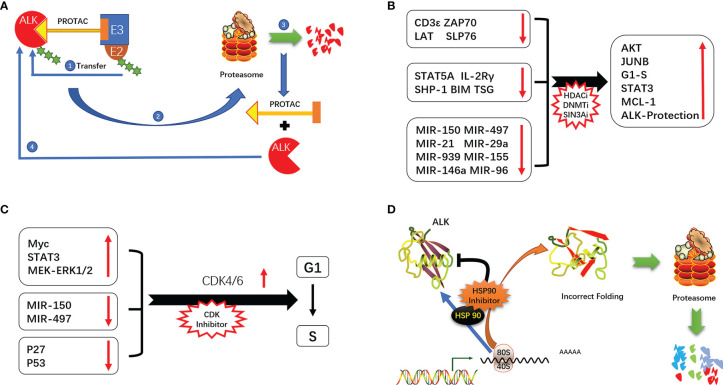
**(A)** Mechanism of action of PROTAC. **(B)** ALK signaling-mediated cycle operation. **(C)** Epigenetic alterations promote the development of ALK-positive ALCL. **(D)** Mechanism of ALK inhibition by HSP90.

PROTACs are used to promote proteasome-mediated protein degradation (111). PROTAC is composed of 2 ligands that connect the target protein and E3 ubiquitin ligase, forming a ternary complex. The ubiquitin-binding enzyme E2 binds to the E2 binding site on E3 ligase and transfers ubiquitin to the target protein, leading to its degradation (111–113). ALK TKIs (alectinib, brigatinib, ceritinib, etc.) are often used as PROTAC ligands to promote the degradation of ALK protein and thereby inhibit tumor growth driven by ALK (114). This allows a small dose of PROTAC to achieve a strong inhibitory effect, which is especially advantageous for overcoming drug resistance due to ALK amplification. PROTAC directly targets and degrades proteins and is unaffected by ALK point mutations; this explains how PROTAC designed with alectinib/brigatinib can degrade the ALK G1202R mutant, which is resistant to the drugs themselves. PROTAC designed with alectinib as the ligand was more effective than alectinib in ALK+ ALCL patients and enhanced ALK degradation (115–117). ARV-110, the first PROTAC drug targeting the androgen receptor, has been used in patients with metastatic debulking-resistant prostate cancer with promising results (118). A second PROTAC drug, ARV-471 (targeting the estrogen receptor [ER]), is being evaluated in clinical trials for the treatment of patients with locally advanced or metastatic ER+/human epidermal growth factor receptor (HER)2− breast cancer. Interim results have shown that ARV-471 reduced ER expression levels by 62% and up to 90% and was effective against both wild-type and mutant ER (119). PROTACs targeting ALK are not yet in clinical use but several ALK-RROTACs are being developed such as SIAIS001, SIAIS117, and SIAIS164018 that can potentially overcome resistance to ALK TKIs (115, 116, 120) (**Figure 4A**).

### 4.4 Targets for Combination Therapy

ALK-independent drug resistance is normally treated by a combination of drugs that inhibit ALK along with other pathways involved in cell survival, thereby enhancing the cytotoxicity of ALK TKIs. The use of ALK TKI combinations can delay the development of ALK resistance. Crizotinib combined with everolimus, CHOP, decitabine, and trametinib prolonged the emergence of resistance, possibly because of a reduction in the dose of individual drugs and the effects of dual targeting (48).

Elevated expression of IL-10RA not only confers resistance to crizotinib but also to second- or third-generation inhibitors. Accordingly, ALK TKI combined with IL-10 pathway inhibitors (e.g., STAT3/pan-JAK/TYK2 inhibitors) may be effective (48) in patients who are resistant to ALK TKIs and have high IL-10RA expression. TYK2 acts upstream of STAT3; it was reported that the survival of ALK+ ALCL cells depended on TYK2/STAT1/MCL1. Regardless of the presence of ALK fusion protein, TYK2 inhibitors can induce tumor cell apoptosis. Inhibiting TY2 blocks the activation of STAT3 by IL-10 and other pathways *via* T2, suppressing tumor cell survival *via* a bypass mechanism (121). The combination of IGF-1R and NPM–ALK inhibited tumor growth in a mouse model of ALCL cell lymphoma. Inhibition of IGF-1R promoted cell apoptosis and blocked phosphorylation of NPM–ALK and its downstream effectors (122). Thus, IGF-1R inhibition combined with crizotinib can have a synergistic effect, allowing dose reduction of crizotinib and delaying the emergence of drug resistance (123). The expression level of platelet-derived growth factor receptor (PDGFR)B in ALK+ ALCL was found to be positively correlated with the cytotoxicity of PDGFR inhibitors, which may be effective in the treatment of PDGFRB+ ALK+ ALCL. Findings from *in vitro* experiments and mouse models have demonstrated that imatinib treatment suppresses the proliferation of tumor cells and promotes apoptosis. Notably, a rapid and durable antitumor response was achieved with imatinib in a patient with advanced refractory ALCL (65). Activation of the MEK pathway was observed in WASP− ALK+ ALCL and was associated with reduced efficacy of ALK TKIs. The combination of the MEK inhibitor trametinib with crizotinib has achieved better clinical outcomes than crizotinib by delaying the emergence of drug resistance (65); thus, targeting MEK can reduce the survival of lymphoma with low or absent WASP expression *via* the MAPK pathway (68). Additionally, γ-secretase inhibitors suppressed the proliferation of ALK+ ALCL cells with crizotinib resistance, suggesting NOTCH1 as a therapeutic target in crizotinib-resistant ALK+ ALCL (124, 125).

Crizotinib combined with everolimus more potently induced cell cycle arrest, DNA damage, and apoptosis than monotherapy in the Karpas299 transplantation model (126, 127). Although everolimus can inhibit mTOR, it can cause the activation of AKT and RAS–ERK, an effect that is blocked by the addition of crizotinib. The combination treatment also prevented the occurrence of selective drug resistance after long-term use of monotherapy (126). In neuroblastoma, crizotinib combined with mTOR inhibitor was shown to overcome crizotinib resistance and promote tumor cell apoptosis (128). In ALK+ ALCL cells, the mTOR inhibitor rapamycin combined with crizotinib increased autophagic flux and promoted cell death (75) (**Figure 2**).

Given the reversibility and importance of epigenetic regulation of gene expression in the development of ALK+ ALCL, drugs targeting epigenetic modifications and allowing “re-expression of tumor suppressor proteins” or “reduced expression of oncogenic proteins”, such as DNA methylation, HDAC and SIN3A inhibitors, are a promising therapeutic approach. (20, 22, 23, 30, 31, 33). In a multicenter clinical study of cidapenem in R/R PTCL, patients with ALK+ ALCL treated with cidapenem had a higher ORR (66.67%) and disease control rate (83.33%) and better prognosis compared to those with other PTCL subtypes (129). The use of decitabine (a DNA methyltransferase inhibitor) not only enhanced the efficacy of ALK TKIs but also prolonged the time to emergence of drug resistance by more than 3 fold compared to monotherapy (48) (**Figure 4B**).

ALK promotes cell cycle progression through a variety of mechanisms (eg, activation of MEK/ERK and STAT3 signaling, induction of cell cycle-related gene expression, and downregulation of P27 and P53) (12, 42, 130, 131). NPM–ALK also promotes cell cycle progression *via* regulation of miRNAs. In ALK+ ALCL, the combination therapy of cell cycle inhibitors has shown the potential of resistance to ALK (132). In a mouse xenograft model of neuroblastoma with ALK F1174L and F1245C mutations, the CDK4/6 inhibitor ribociclib blocks the binding of CDK4/6 to CyclinD1, thereby inhibiting the operation of the cell cycle. In combination with Ceritinib exerts synergistic cytotoxicity, inhibits tumor growth, enhances cycle arrest, and promotes cell death (133) (**Figure 4C**).

### 4.5 Targets Other Than ALK

ALK+ ALCL expresses CD30, PD-L1, and B7-H3, which are potential therapeutic targets for promoting tumor cell death through pathways independent of ALK, thereby overcoming ALK TKI resistance.

#### 4.5.1 CD30

ALK promotes CD30 expression through the MEK–ERK–AP1–JUNB pathway (134, 135). Brentuximab vedotin (BV) is an antibody–drug conjugate (ADC) that targets CD30 in which anti-CD30 antibody is linked to the microtubule destroyer monomethyl auristatin E (MMAE) *via* a protease-cleavable linker. After binding to CD30, BV forms phagosomes through receptor-mediated endocytosis before it is hydrolyzed by lysosomal proteases; this releases MMAE, leading to cell cycle arrest and cell apoptosis (136–139). At the 5-year follow-up of a clinical trial of BV in R/R ALCL, 66% of patients continued to show a CR (140). In another study of BV in the treatment of R/R ALCL, 86% of patients achieved ORR including 57% with CR and 29% with partial remission; the duration of response was 12.6 months and the duration of CR was 13.2 months (141). In a clinical trial of BV combined with chemotherapy in the treatment of pediatric patients with first-onset ALK+ ALCL (NCT01979536), the 2-year EFS rate was 79.1% and 2-year OS rate was 97.0% (142). Only one patient (1.5%) relapsed during treatment. These results demonstrate that BV is superior to conventional chemotherapy in preventing recurrence.

In a clinical trial of chimeric antigen receptor (CAR) T-cell (CAR-T) therapy targeting CD30 in ALCL patients (NCT01316146), the duration of CR was up to 9 months after 4 infusions. Importantly, CD30 CAR-T was still detectable after 6 weeks of treatment, indicating that CAR-T had sustained antitumor effects. ADCs targeting CD30 are associated with a number of adverse effects including gastrointestinal reactions that reduce the tolerability of these drugs and can lead to treatment discontinuation. CD30 CAR-T therapy circumvents this problem and under standardized care and execution, is a safe and effective treatment for CD30+ lymphoma (143).

#### 4.5.2 HSP90

HSP90 is highly expressed in tumors including ALK+ ALCL. HSP90 inhibitors not only block the binding of HSP90 to ATP but also promote proteasome-mediated degradation of HSP90 target proteins (144). Inhibition of HSP90 resulted in the downregulation of NPM–ALK. The combined use of the HSP90 inhibitor onalespib and ALK TKI in ALK+ NSCLC delayed the emergence of ALK resistance and preserved sensitivity to onalespib (145). A dual-target inhibitor designed based on the active structure of HSP90 and ALK TKIs (resorcinol and 2,4-diaminopyrimidine motifs) has been proposed that is expected to overcome ALK TKI resistance (146) (**Figure 4D**).

#### 4.5.3 Immune Checkpoint

In ALK+ ALCL, whether NPM-ALK promotes PD-L1 expression through downstream signals or in view of the role of PD-1/PD-L1 in TME, PD1/PD-L1 is expected to become a potential therapeutic target for ALK+ ALCL. Recently, studies have found that the expression of PD-L1 and the number of tumor-infiltrating T cells are related to the prognosis of ALK+ ALCL (147). Geptanolimab (GB226) is a PD-1 monoclonal antibody. In an open study (NCT03502629), it was found that the higher the level of PD-L1 in R/R PTCL, the effect of PD-1 blockers is better. In R/R PTCL, the ORR was 40.4% and the 12-month Duration of Response (DOR) was 48.5%. In PTCL with PD-L1 expression>50%, the ORR (53.3%) and median PFS (6.2 months) were higher, especially in ENKTL, ALK^-^ALCL, ALK^+^ALCL, Geptanolimab has a better curative effect (148). Similar to this, in an ALK-resistant ALK+ ALCL patient whose tumor tissue highly expresses PD-L1, the tumor tissue completely disappeared after 5 months of Navumab treatment and the complete remission was maintained for up to 18 months (149). In addition, when B7-H3 CAR-T is used to treat ALK+ ALCL, it shows strong cytokine secretion and cytotoxicity both in *in vivo* and *in vitro* experiments. It has obvious proliferative activity and memory phenotype after receiving B7-H3 stimulation. Under the premise of strict control of CRS, B7-H3 CAR-T is expected to become another important therapeutic target for ALK+ ALCL after ALK and CD30 (150).

## 5 Discussion

ALK TKI resistance is a major challenge in the treatment of ALK+ ALCL; therefore, therapeutic strategies to overcome this resistance are a key research direction for this malignancy. Many aspects of the resistance mechanisms remain to be elucidated including the upregulation of Bcl-2, ALK amplification after the application of crizotinib, bypass signaling, autophagy, and apoptosis. There have been few studies of single drugs/combination therapies that can improve/reverse ALK resistance, which may be related to the low prevalence of ALK+ ALCL and do not provide sufficient impetus for clinical trials. There are also considerable disparities in the treatment of ALK TKI resistance, especially in terms of therapeutic options for patients. Strategies that target resistance mutations are expected to greatly improve the clinical outcome of ALK+ ALCL.

Selection of appropriate ALK TKIs by sequencing ALK mutation sites can alleviate ALK TKI resistance, although highly resistant mutants and multiple mutations are problematic. The mechanisms by which mutations lead to ALK resistance have been systematically investigated in studies of ALK protein structure and binding to TKI and ATP, and they have served as the basis for the evaluation of candidate drugs such as ZX-29 and ginitinib. The latter in particular—whose binding to ALK is largely unaffected by ALK mutations—can overcome the effects of most ALK single mutations as well as double mutations conferring loratanib resistance (99, 106, 107).

ALK amplification also underlies resistance to ALK TKIs (57) although it may not benefit tumor cells as it can trigger oncogenic stress and induce DNA damage especially upon discontinuation of an ALK TKI, resulting in tumor cell apoptosis and restoration of sensitivity to the inhibitor (85). PROTAC technology is a new treatment strategy that targets ALK amplification and is effective against drug resistance mutations as it induces mutant ALK proteins to undergo ubiquitin-mediated degradation (114).

ALK-independent drug resistance usually necessitates treatment with a combination of drugs, and many studies have shown that although ALK inhibition decreases tumor cell survival and proliferation, many cellular changes caused by ALK overexpression are not reversed such as IL-10RA and IGF-1R dysregulation (59, 62–64, 68). When the inhibition of ALK fusion proteins is alleviated, these changes allow tumor cells to survive and proliferate, leading to the development of ALK TKI resistance. Pro-survival pathways other than ALK can be blocked by a combination of drugs that synergistically enhance the cytotoxicity of ALK TKIs. Additionally, drug combinations can also delay the emergence of resistance through dose reduction of single agents and dual targeting. Although there have been only a few clinical studies examining ALK TKI combinations in ALK+ ALCL, clinical trials are currently underway (**Table 2**). ALK TKIs also cause adaptive changes in tumor cells that favor their survival such as the upregulation of Bcl-2 and inhibition of autophagy; therapeutic strategies that target these changes may be effective in the treatment of ALK+ ALCL (56, 77, 79).

**Table 2:** Recruiting ALCL clinical trials.

Study Title	Recruiting clinical trials	Phase	Note
Lorlatinib	NCT03505554	2	ALK+ALCL
Nivolumab	NCT03703050	2	R/R ALK+ ALCL
CD30 Targeted CAR-T	NCT03383965	1	HL、ALCL
	NCT04526834	1	ALCL、PTCL DLBCL 、ENKTL PMBCL
	NCT04008394	1	HL、ALCL PTCL、NKTCL
BV+Chemotherapy	NCT03113500	2	CD30+PTCL
Lenalidomide+CHOP	NCT04423926	1/2	Untreated PTCL
Venetoclax+Romidepsin	NCT03534180	2	R/R Mature T-Cell Lymphoma
Crizotinib+ Etoposide Capsule	NCT03707847	4	R/R ALK+ ALCL
Pembrolizumab+Pralatrexate	NCT03598998	1/2	R/R PTCL
Pembrolizumab+Romidepsin	NCT03278782	1/2	R/R PTCL

In addition to ALK fusion proteins and their associated signaling pathways, activation of ALK and its downstream effectors in ALK+ ALCL results in the expression of CD30, PD-L1, and B7-H3, which are potential drug targets in patients who are highly resistant to ALK TKIs. However, it is unclear whether such targeted therapies can restore tumor cell sensitivity to the inhibitors.

## 6 Conclusion

In this review, we summarized research progress on ALK resistance to provide a reference for the design of clinical studies and development of new drugs for the treatment of ALK+ ALCL. ALK is an important therapeutic target in ALK+ ALCL. ALK TKIs have broadened the therapeutic options for ALK+ALCL patients who are resistant to or relapse on chemotherapy, but the emergence of drug resistance is an outstanding problem. Considerable progress has been made in the elucidation of ALK resistance mechanisms including those associated with and independent of ALK, and novel TKIs are being developed that can bring lasting remission to ALK TKI-resistant patients.

## Author Contributions

YW and JH performed the analysis and wrote the manuscript. MX and QX checked and embellished the language. CZ searched the relevant literature and drew the figures. WS and YZ designed the ideas for the article. All authors contributed to the article and unanimously agreed to submit the review.

## Funding

This study was supported by the National Natural Science Foundation international cooperation (81570184), the Science and Technology Project of Nantong City (MS22018008), the Science and Technology Project of Nantong City (MS12017003-2), China Postdoctoral Science Foundation (2019M660127), Jiangsu Province Postdoctoral Science Foundation (2019K062), Jiangsu Province Postdoctoral Foundation (2019Z146), the Municipal Natural Science Foundation of Nantong (Nos. JCZ20207).

## Conflict of Interest

The authors declare that the research was conducted in the absence of any commercial or financial relationships that could be construed as a potential conflict of interest.

## Publisher’s Note

All claims expressed in this article are solely those of the authors and do not necessarily represent those of their affiliated organizations, or those of the publisher, the editors and the reviewers. Any product that may be evaluated in this article, or claim that may be made by its manufacturer, is not guaranteed or endorsed by the publisher.
